# Effects of intestinal colonization by *Clostridium difficile* and *Staphylococcus aureus* on microbiota diversity in healthy individuals in China

**DOI:** 10.1186/s12879-018-3111-z

**Published:** 2018-05-03

**Authors:** Danfeng Dong, Qi Ni, Chen Wang, Lihua Zhang, Zhen Li, Cen Jiang, EnqiangMao, Yibing Peng

**Affiliations:** 1 Department of Laboratory Medicine, Ruijin Hospital, Shanghai Jiaotong University School of Medicine, No.197 Ruijin ER Road, Shanghai, 200025 China; 2 Department of Laboratory Medicine, Xinhua Hospital, Shanghai Jiaotong University School of Medicine, No. 1665 Kongjiang Road, Shanghai, 200092 China; 3 Department of Laboratory Medicine, Longhua Hospital, Shanghai University of Traditional Medicine, No725 South Wanping Road, Shanghai, 200032 China; 4 Department of Emergency Intensive Care Unit, Ruijin Hospital, Shanghai Jiaotong University School of Medicine, No.197 Ruijin ER Road, Shanghai, 200025 China

**Keywords:** *Clostridium difficile*, *Staphylococcus aureus*, Intestinal colonization, Gut microbiota

## Abstract

**Background:**

Intestinal colonization by pathogenic bacteria is a risk factor for infection, and contributes to environmental contamination and disease dissemination. Alteration of gut microbiota also plays a pivotal role in the development of disease. Although *Clostridium difficile* and *Staphylococcus aureus* are well-recognized pathogens causing nosocomial and community infections, the intestinal colonization was not fully investigated. Herein, we explored their overall carriage rates in healthy adults from the community, and characterized the gut microbiomes of *C. difficile* and *S. aureus* carriers.

**Methods:**

Fecal samples were collected from 1709 healthy volunteers from communities in Shanghai, China, and tested for the presence of *C. difficile*, methicillin-sensitive *S. aureus* (MSSA), and methicillin-resistant *S. aureus* (MRSA) using culture-based techniques. To explore differences in the gut microbiome, 16S rRNA gene sequencing was conducted using samples from non-carriers (CH), *C. difficile* carriers (CCD), MRSA carriers (CM), and MSSA carriers (CS).

**Results:**

Overall, we detected 12 *C. difficile* and 60 *S. aureus* isolates, accounting for 0.70% and 3.51% of total isolates, respectively. Eight isolates were determined to be MRSA, accounting for 13.3% of the *S. aureus* population. Sequencing data revealed that the microbial diversity and richness were similar among the four groups. However, at the phylum level, carriage of *C. difficile* or MRSA was associated with a paucity of *Bacteroidetes* and an overabundance of *Proteobacteria* compared with non-carriers. At the genus level, the prevalence of the genera *Bacteroides*, *Prevotella*, *Faecalibacterium*, and *Roseburia* was decreased in *C. difficile*-positive samples compared with the controls, while the proportion of *Clostridium* cluster XIVa species was increased. MRSA carriers exhibited a higher proportion of the genera *Parasutterella* and *Klebsiella*, but a decreased prevalence of *Bacteroides*. Compared with MSSA carriers, *Klebsiella* was the only genus found to be significantly enriched in MRSA carriers.

**Conclusions:**

In healthy adults, colonization by *C. difficile* or *S. aureus* did not significantly affect gut microbiota diversity. However, the alteration of the gut microbiota composition in *C. difficile* carriers could indicate a predisposition to further infection. Our study provides essential data on the prevalence and effects of *C. difficile* and *S. aureus* colonization on gut microbiota composition in healthy adults.

**Electronic supplementary material:**

The online version of this article (10.1186/s12879-018-3111-z) contains supplementary material, which is available to authorized users.

## Background

*Clostridium difficile* and *Staphylococcus aureus* are well known pathogens, causing both nosocomial and community-acquired infections. Although the clinical manifestations of these pathogens are diverse, their colonization and subsequent dissemination from the intestinal tract show a commonality [1, 2]. *C. difficile* is widely accepted as the most common cause of antibiotic-associated diarrhea, and the incidence and severity of *C. difficile* infection has dramatically increased over past decades [3]. Unlike *C. difficile*, *S. aureus* gut colonization, which is a known risk factor for gastro-intestinal infection [4], has not yet been well studied. During the past decade, with the pandemic rise in the incidence of methicillin-resistant *S. aureus* (MRSA) and methicillin-sensitive *S. aureus* (MSSA) infection in the community and in healthcare facilities, fecal carriage of *S. aureus* has attracted attention. For example, studies have shown that patients with intestinal colonization of MSSA and/or MRSA have a higher frequency of diarrhea and environmental contamination, as well as increased skin colonization [4, 5]. While many studies have investigated the current status of *C. difficile*, MSSA, and MRSA colonization of high-risk populations and healthcare facilities, there is limited data regarding the prevalence and colonization of these pathogens in healthy individuals in the community in China.

The diverse and abundant microbiota of the human intestine plays a crucial role in protection against pathogen colonization, as well as in overall human health [6]. However, intestinal infection or colonization by pathogens such as *S. aureus* and *C. difficile* is associated with disruption of the gut microbiota [7]. Numerous studies have shown that the biodiversity and community structure of the gut microbiota is altered in patients infected with *C. difficile* or *S. aureus*, and these alterations may directly lead to diseases [6, 8]. In addition, gut dysbiosis caused by MRSA colonization is more severe than that caused by MSSA, with reduced microbial diversity and a decreased prevalence of beneficial bacterial [9]. Fecal microbiota transplantation has been successfully used to treat *C. difficile* infection and MRSA-associated enterocolitis [10], indicating that modulation of the gut microbiota may be a potentially useful approach to treat refractory infections. However, whether the intestinal bacterial communities of asymptomatic carriers are notably changed in individuals after joining a community has not yet been studied and needs to be clarified.

The main focus of this study was to estimate the prevalence of *C. difficile* and *S. aureus* in the gut of healthy adults in the communities, and to investigate whether the carriage of these pathogens was related to alterations in the gut microbiota composition.

## Methods

### Study design and specimen collection

The present study was conducted in four communities in Shanghai, China, between May and August 2014. Healthy individuals aged over 18 years without any acute or chronic gastrointestinal diseases were enrolled. Those who were exposed to any hospital environment or antibiotics in the previous 30 days before collection were excluded. Demographic data concerning the age and gender of the participants was collected. A total of 1709 participants were enrolled in the present study. Among them, 748 were female and 961 were male. The average age was 63 (+/− 16) years old. To better document age-related kinetics, we categorized the participants into three groups based on their ages: youth (18–40 years), middle-aged (41–65 years), and elderly (> 65 years). Fresh fecal samples were collected into sterile containers and submitted for immediate culture. All samples were stored at − 80 °C for subsequent DNA extraction.

To further characterize the intestinal microbiota of *C. difficile*- and *S. aureus*-positive individuals, we divided participants into four groups: 1) healthy individuals without any *C. difficile* or *S. aureus* colonization (group CH), 2) individuals positive for *C. difficile* (group CCD), 3) individuals positive for MRSA (group CM), and 4) those positive for MSSA (group CS). Due to the results that there were fewer individuals positive for *C.difficile* and MRSA, all individuals meeting the criteria for the CCD and CM groups were included in the study, with participants in the CH and CS groups randomly chosen and then age and gender-matched with participants in the first two groups. Detailed information regarding the selected participants is listed in Additional file 1: Table S1.

### Detection of bacterial pathogens

To improve the sensitivity of *C. difficile* detection, we aliquoted each stool sample into two parts. One aliquot was pretreated with ethanol and then directly plated onto cycloserine cefoxitin fructose agar (Oxoid Ltd., Basingstoke, UK). The other was pre-cultured in cycloserine cefoxitin fructose medium supplemented with taurocholic acid and lysozyme for 48 h to enrich the vegetative cells prior to plating on cycloserine cefoxitin fructose agar. Culturing was performed at 35 °C for 48 h in anaerobic condition. Suspected *C. difficile* colonies were identified based on their odor and appearance, and confirmed using a latex agglutination test (*C. difficile* Agglutination Test Kit; Oxoid), *gluD* gene detection, and a toxin A & B test using a VIDAS Immunoanalyzer (Biomerieux, Marcy-l’Etoile, France).

To screen *S. aureus* isolates from stool samples, samples were plated on mannitol salt agar (Becton Dickinson) and cultured for 48 h at 35 °C in aerobic condition. Suspected *S. aureus* colonies were identified based on microbiological examination. MRSA was further confirmed by Kirby-Bauer disk diffusion assay (30 mg/liter cefoxitin) [11] and *mecA* gene detection using *S. aureus* ATCC25923 as a negative control.

### DNA extraction

Approximately 200 mg of each fecal sample was used for genomic DNA extraction using a TIANamp Stool DNA Kit (Tiangen Biotech, Beijing, China) as per the manufacturer’s instructions. A NanoDrop spectrophotometer (Thermo Fisher Scientific, Waltham, MA, USA) was used to measure the DNA concentration and A260/A280 ratio. A PCR assay targeting the V3–V4 regions of the 16S rRNA gene was then conducted using universal primers (F: 5′-GAGAGTTTGATCCTGGCTCAG-3′; R: 5′-AAGGAGGTGATCCAGCCGCA-3′) to further confirm the quality and quantity of extracted DNA.

### 16S rRNA gene sequencing and data processing

16S rRNA gene amplification and sequencing was conducted in the Chinese National Human Genome Center (Shanghai, China). Briefly, hyper variable V3–V4 regions of the 16S rRNA genes were amplified using primers V3-343F (5′-TACGGRAGGCAGCAG-3′) and V4-798R (5′-AGGGTATCTAATCCT-3′) with adaptor sequences (LinkerF: 5′-TCGTCGGCAGCGTCAGATGTGTATAAGAGACAG-3′; LinkerR: 5′-GTCTCGTGGGCTCGGAGATGTGTATAAGAGACAG-3′), followed by sequencing using the Illumina Miseq system via standard procedures. Sequencing reads were screened to remove low quality reads, unmatched barcode sequences, and short read lengths.

Quality-trimmed sequences were clustered into one operational taxonomic unit (OTU) at a 97% similarity cutoff. Taxonomic assignment was carried out using the Ribosomal Database Project Naïve Bayesian Classifier with a cutoff value of 0.03. The rarefaction curves were measured at an OTU level of 0.03 to evaluate the abundance between samples. Alpha diversity assessment, including community richness (abundance-based coverage estimator (ACE) richness index and Chao richness index analyses) and community diversity (Shannon diversity index and Simpson diversity index analyses), was carried out using mothur software (available from https://www.mothur.org/).

### Statistical analyses

Differences in biodiversity index between four groups were compared using a one-way analysis of variance test. To analyze the relative intestinal composition at the phylum and genus level between groups, a Mann-Whitney test was conducted in GraphPad Prism 5. All differences were considered significant at *P* < 0.05.

## Results

### Study population

As shown in Tables 1, 12 *C. difficile* and 60 *S. aureus* isolates were identified, accounting for 0.70% and 3.51% of the total number of isolates, respectively. Of the *S. aureus* isolates, eight (13.1%) were confirmed to be MRSA. None of the participants were found to be colonized with both *C. difficile* and *S. aureus*. Compared with the two younger groups, the elderly participants were more frequently positive for *C. difficile*. However, the prevalence of *S. aureus* decreased with age, with the highest prevalence (6.15%) observed in youth, and the lowest (2.7%) prevalence in the elderly (Table 1).

**Table 1:** Prevalence of *Clostridium difficile* and *Staphylococcus aureus* in the intestines of healthy adults in the community in Shanghai, China, 2014

Age (years)	No.	*C. difficile*		*S. aureus*	
		No.	%	No.	%
18–40	130	0	0.00%	8	6.15%
41–65	837	3	0.36%	32	3.82%
> 65	742	9	1.21%	20	2.70%
Overall	1709	12	0.70%	60	3.51%

### Sequencing reads and biodiversity

**Fig. 1:**
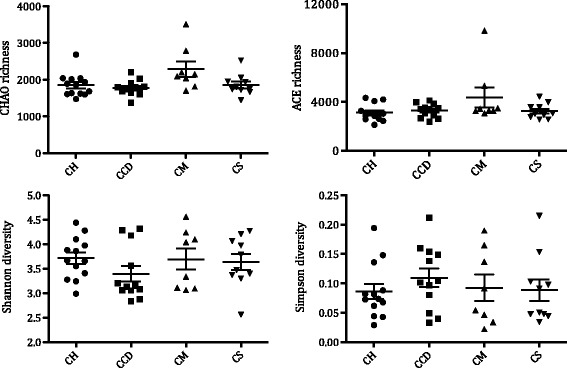
Indices of microbial richness and diversity for all samples (shown in group). Data represent the mean value and standard error of each group. CH: non-carriers; CCD: *Clostridium difficile* carriers; CM: methicillin-resistant *Staphylococcus aureus* carriers; CS: methicillin-sensitive *Staphylococcus aureus* carriers

We obtained a total of 1,404,279 valid sequences from the 43 selected participants after barcode trimming and primer sequencing, with an average of 32,657 sequences per sample. Subsequent analysis was performed after normalizing the sequence depth of all samples to that of the lowest sample size (12,394). All samples had good index coverage (> 95%), with OTU-level rarefaction curves almost reaching a plateau, indicating sufficient sequencing depth (Additional file 2: Figure S1a). The number of OTUs for each sample ranged from 369 to 900, with similar median numbers of OTUs across the four groups (Additional file 2: Figure S1b). Compared with the healthy controls, the *C. difficile*-positive samples had a lower average Shannon diversity index value and a higher average Simpson diversity index value; however, these differences were not significant, even when community richness was assessed using Chao and ACE index analyses (Fig. 1). The median community richness and diversity values for the MSSA- and MRSA-positive samples were similar to those of the healthy controls (Fig. 1).

### Microbial community structure

**Fig. 2:**
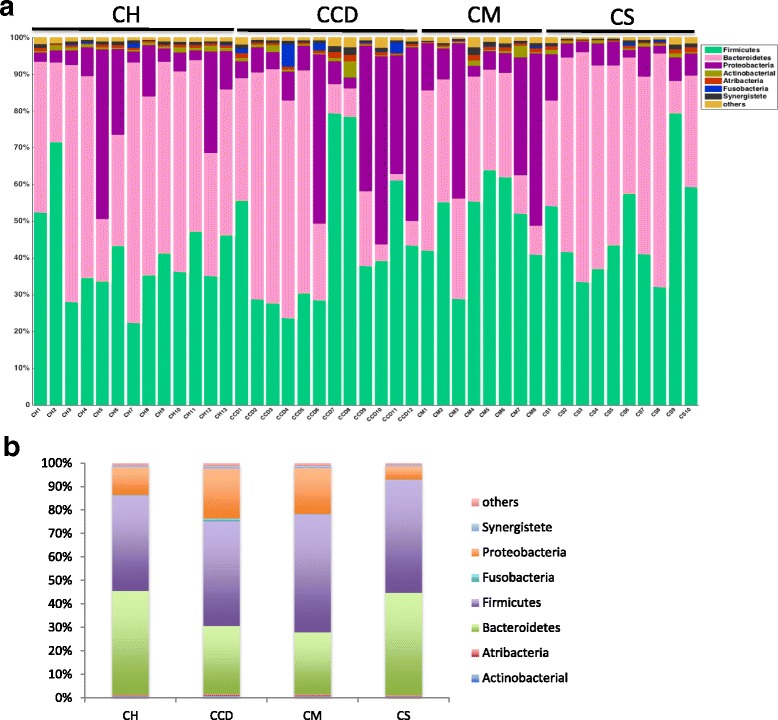
**a** Microbial composition of each sample in the different groups at the phylum level. **b** Relative proportions of phyla in each group. Phyla are shown as the percentage of total operational taxonomic units. CH: non-carriers; CCD: *Clostridium difficile* carriers; CM: methicillin-resistant *Staphylococcus aureus* carriers; CS: methicillin-sensitive *Staphylococcus aureus* carriers

The distribution of the most dominant bacterial phyla in each of the groups is listed in Fig. 2a. Although there was variation among all samples, *Firmicutes* and *Bacteroidetes* were always the predominant bacterial phyla. When analyzing the pooled samples in each group, we clearly observed that compared with the healthy controls, the CCD and CM groups had a noticeably lower proportion of *Bacteroidetes* (28.98% and 26.47% respectively versus 44.20% of the controls) but a relatively higher proportion of *Proteobacteria* (21.36% and 19.51% respectively versus 11.80% of the controls) (Fig. 2b). The distribution of bacterial phyla in the CS group was similar to that of the healthy controls, except for a decrease in the prevalence of *Proteobacteria* (5.61%) (Fig. 2b).

**Fig. 3:**
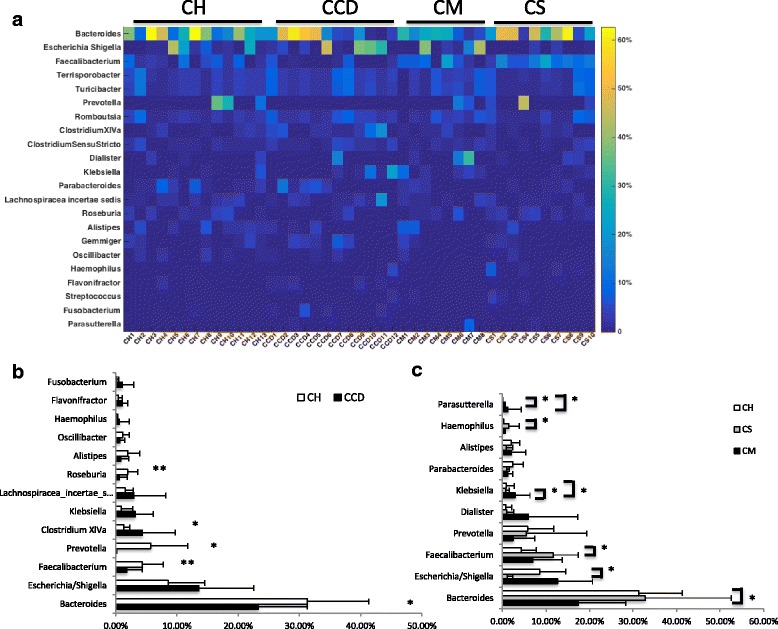
**a** Heatmap showing the relative percentages of the most abundant genera in each sample within the different groups. **b** Relative percentages of the bacterial genera in groups CH and CCD. **c** Relative percentages of the bacterial genera in groups CH, CM, and CS. CH: non-carriers; CCD: *Clostridium difficile* carriers; CM: methicillin-resistant *Staphylococcus aureus* carriers; CS: methicillin-sensitive *Staphylococcus aureus* carriers. *, *P* < 0.05; **, *P* < 0.01

At the genus level, all genera present at a relative abundance of > 1% are shown in Fig. 3a. In individuals positive for *C. difficile*, we observed significant decreases in the prevalence of *Bacteroides* and *Prevotella* species within the phylum *Bacteroidetes*. The abundance of *Prevotella* in the CCD group sharply declined from 5.33% to 0.03%. *Firmicutes*, *Faecalibacterium*, and *Roseburia* sequences were found to decrease in prevalence in the CCD group, while there was a significant increase in the prevalence of *Clostridium* cluster XIVa. Although there were mean differences in the proportions of *Escherichia/Shigella*, *Klebsiella*, *Lachnospiracea incerae sedis*, *Alistipes*, and *Fusobacterium* in the gut microbiota of the CCD group, no statistical significance was found (Fig. 3b).

Surprisingly, the composition of the gut microbiota of MRSA-positive samples differed markedly from that of the MSSA-positive samples (Fig. 3c). MSSA-positive samples had a relative higher proportion of *Faecalibacterium*, *Parabacteroides*, and *Haemophilus*, but a lower percentage of *Escherichia/Shigella*. In comparison, the MRSA-positive samples had a significantly higher prevalence of *Klebsiella* and *Parabacteroides* (*P* < 0.05), but a lower proportion of *Bacteroides*. It is also noteworthy that the relative abundance of *Parasutterella* species increased progressively from the CH to the CS samples and then to the CM samples. *Klebsiella* was the only genus that was significantly enriched in the MRSA-positive samples compared with the MSSA-positive samples.

Principal coordinate analysis of unweighted UniFrac distances was used to identify the potentially divergent clusters among different groups based on the microbiota structure. However, the plot showed a moderate to high degree of overlap, and failed to discriminate any differences between groups (Additional file 2: Figure S2).

## Discussion

According to reports from China, the asymptomatic colonization with *C.difficile* was estimated to 10–20% in hepatic cirrhosis or preoperative colorectal cancer patients [12, 13]. However, to our best knowledge, the colonization pattern of *C.difficile* in healthy individuals has not been well determined domestically. In the present study, we found that the gut colonization rate of *C. difficile* among healthy adults in the community was 0.7%. This was in agreement with a report from Hesse, Germany [14], which identified a *C. difficile* colonization rate of 0.8% in healthy adults living outside long-term care facilities. However, the age structure of the two studies differed, with an average age of participants in the current study of 63 years, while that of the previous study was 48 years. As both studies showed that the acquisition of *C. difficile* increased with age, the overall colonization rate in Shanghai, China might be relatively lower than that in Hesse, Germany. Among the elderly, our observed colonization rate of 1.35% was similar to that reported by Rea et al. in Ireland [15], but lower than a previous report of 4% in community-dwelling elderly people in the United Kingdom [16]. As no outbreaks of *C. difficile* infection have yet occurred in Shanghai, it is reasonable to conclude that the colonization rate is not as high as that in European countries. However, because of the population pyramid structure and the ageing population in China, it will be important to monitor *C. difficile* colonization and infection rates, especially in community settings.

Numerous studies have shown that nasal carriage of *S. aureus*, the most common site of colonization, is a risk factor for infection [17]. However, since the epidemic-scale emergence of community-acquired MSSA and MRSA, several extra-nasal carriage sites have aroused attention. Intestinal colonization is a vital concomitant reservoir for *S. aureus*, but is poorly understood. In the present study, the overall prevalence of *S. aureus* is the gut was 3.51%, with MSSA and MRSA accounting for 3.04% and 0.47% of all isolates, respectively. According to the meta-analysis conducted by Gagnaire et al. involving 712 studies [18], the intestinal carriage rate in healthy adults was estimated to be approximately 13.8% for *S. aureus* and 1.4% for MRSA, much higher than those detected in the current study. The studies included in the meta-analysis were mostly focused on specific populations at high risk of *S. aureus* carriage, rather than those strictly defined as healthy community-based individuals. Regardless, it is noteworthy that the proportion of MRSA among the total *S. aureus* population in the current study was 13.33%, which was similar to the 10.14% reported in the meta-analysis. Previous studies have shown that *S. aureus* can colonize the human intestine at any ages from birth, but that the colonization frequency appears to drop later in the life [19]. However, no consensus on this observation has been achieved. Herein, we found that the *S. aureus* carriage rates declined with age, with the prevalence rates in youth twice as high as those in the elderly. This trend was in complete contrast to the colonization pattern observed for *C. difficile*. However, the association between *S. aureus* carriage and age needs to be further investigated.

The human gut is a complex ecosystem inhabited by trillions of commensal bacterial, providing a healthy microenvironment for uptake of essential nutrients, metabolism, and immune protection, as well as pathogen resistance [20]. Alterations in the normal intestinal microbiota often impair colonization resistance, thus allowing for the establishment and proliferation of enteric pathogens such as *C. difficile* and *S. aureus* [8]. Microbial communities in *C. difficile*-positive and MRSA-infected patients from healthcare facilities always show a marked reduction in bacterial richness and diversity [21]. However, the intestinal microbiota of hospitalized patients can be influenced by various factors, including antibiotics and underlying disease. In our study, in healthy individuals from the community, we did not observe any significant changes in species diversity or richness in the gut microbiota of *C. difficile* or *S. aureus* carriers compared with the controls, and no differences in community structure were found by principal coordinate analysis. These findings suggested that colonization by the two pathogens did not significantly alter the microenvironment balance. Host factors such as immune status or comorbidities may affect the integrity of the intestinal microbiota, which could potentially explain the different findings regarding microbial richness in hospitalized and community-based populations.

Although the species richness and diversity in the intestines of the *C. difficile* and *S. aureus* carriers were similar to those of non-carriers, there were differences in the community composition between the carriers and non-carriers. Imbalances in the composition of commensal bacterial populations can predispose individuals to infection inflammatory diseases, including inflammatory bowel disease [22]. In accordance with findings regarding *C. difficile* infection and asymptomatic patients by Zhang et al. [21], we observed a lower level of *Bacteroidetes* and a higher prevalence of *Proteobacteria* in *C. difficile* and MRSA carriers, implying that species belonging to these two bacterial phyla are sensitive to the presence of *C. difficile* and MRSA, but not MSSA. Interestingly, eradication of *Proteobacteria* and restoration of *Bacteroidetes* are considered predictors for successful fecal microbiota transplant [23], emphasizing the importance of balance amongst commensal bacteria. Infection or colonization with *C. difficile* is usually accompanied by depletion or expansion of numerous species related to protein digestion or inflammatory regulation, triggering the overgrowth of enteric pathogens [24]. In our study, the gut populations of *C. difficile* carriers showed a depletion of butyrate-producing bacterial genera *Roseburia* and *Faecalibacterium*, as well as *Bacteroidetes* genera *Bacteroides* and *Prevotella*. Butyrate is an energy source for colonocytes, and as such plays a pivotal role in the regulation of colonocyte differentiation, which is important in maintaining intestinal epithelial integrity [25]. Thus, butyrate-producing bacteria may enhance colonic defense barriers by producing antimicrobial peptides, protecting the host from infection. The observed paucity of *Roseburia* and *Faecalibacterium* in healthy *C. difficile* carriers in the current study implies that these two genera could be used to represent *C. difficile*-sensitive butyrate-producing bacteria. Like butyrate-producing bacteria, *Bacteroides* and *Prevotella* species, which are frequently decreased in patients with *C. difficile* infection or colonization, protect the host against pathogen-mediated colitis inflammation by digesting carbohydrates and producing essential substrates for colonocytes [26]. However, despite only a small subset of genera being affected by *C. difficile* colonization in healthy community-based individuals, our findings did suggest that the gut microbiota of *C. difficile* carriers was more susceptible to further infection.

The gut microbiota signatures of *S. aureus* carriers have not been well determined. Our findings showed that dysbiosis was not present in the gut microbiota of MSSA carriers with regards to either community biodiversity or composition at the phylum level. Although some genera showed differences in, e.g., *Haemophilus*, *Faecalibacterium*, and *Escherichia/Shigella*, which might be related to the presence of MSSA, we could not define the specific role of the changes in terms of microbiota status. Zhao et al. showed that patients with MRSA infection had specific gut microbiota features, exhibiting reduced diversity and decreased prevalence of short-chain fatty acid producers compared with MRSA-negative patients [9]. In our group of healthy community-based adults, the reduction of *Bacteroides* species in MRSA carriers was in accordance with the previous report, but no alteration in the prevalence of butyrate-producers was observed. However, the progressively increased proportion of *Parasutterella* species in MSSA and then MRSA samples was noteworthy. *Parasutterella* (phylum *Proteobacteria*) is a relatively recently described genus that is enriched in individuals with Crohn’s disease [27]. *Klebsiella*, another opportunistic enteric bacterial genus, was the only genus showing increased prevalence in MRSA carriers than in MSSA carriers. Antibiotic usage is reported to significantly enhance the proportion of *Klebsiella* in the gut [28], indicating that co-colonization by MRSA and *Klebsiella* might be related to exposure to antibiotics in the gut microenvironment. However, because of the small sample size used in the present study, more research needs to be carried out to identify specific genera associated with MRSA or MSSA carriage.

## Conclusion

In conclusion, this study examined the overall prevalence of *C. difficile*, MSSA, and MRSA in healthy community-based adults in China, providing basic epidemiological data concerning these clinically important pathogens. In this healthy population, the presence of *C. difficile* or *S. aureus* did not significantly alter the community diversity or richness, with only a small set of genera showing changes in prevalence. However, *C. difficile* carriers exhibited a decrease in the prevalence of *Bacteroidetes* and butyrate-producing genera, indicating a predisposition to further infection. In addition, the specific role of the altered genera in MRSA- and MSSA-positive samples should be further investigated. Taken together, our findings would help to better understand the characteristics of gut microbiota in *C. difficile* and *S. aureus* carriers in a community setting, providing necessary data for future surveillance and furthering our knowledge of the development of infection.

## Additional files

Additional file 1: **Table S1.** Detailed information on participants selected for microbiota analysis. (DOCX 42 kb)

Additional file 2: **Figure S1.** Rarefaction curves for each sample and OTU numbers of each group. **Figure S2.** Principal coordinate analysis (PCoA) of bacterial communities using unweighted UniFrac distances of 16S rRNA gene sequences. (PPTX 521 kb)

## Abbreviations

MRSA: Methicillin-resistant *S. aureus*
MSSA: Methicillin-sensitive *S. aureus*
OTU: Operational taxonomic unit
