# Supplementary material for: Effects of intestinal colonization by Clostridium difficile and Staphylococcus aureus on microbiota diversity in healthy individuals in China
Source: BMC Infect Dis. 2018 May 3;18:207. doi: 10.1186/s12879-018-3111-z (PMC5934869; doi:10.1186/s12879-018-3111-z)
Supplement: Supplementary file 1 — Table S1. Detailed information on participants selected for microbiota analysis. (DOCX 42 kb) [file 12879_2018_3111_MOESM1_ESM.docx]

Table S1 Detailed information on participants selected for microbiota analysis

| Group | No. | Gender | Age |
| --- | --- | --- | --- |
| CH | 1 | M | 74 |
|  | 2 | M | 84 |
|  | 3 | M | 64 |
|  | 4 | M | 86 |
|  | 5 | M | 52 |
|  | 6 | F | 89 |
|  | 7 | M | 90 |
|  | 8 | M | 74 |
|  | 9 | M | 89 |
|  | 10 | M | 35 |
|  | 11 | M | 39 |
|  | 12 | F | 58 |
|  | 13 | F | 33 |
| CCD | 1 | M | 76 |
|  | 2 | M | 75 |
|  | 3 | M | 76 |
|  | 4 | M | 75 |
|  | 5 | M | 82 |
|  | 6 | M | 83 |
|  | 7 | M | 86 |
|  | 8 | M | 48 |
|  | 9 | F | 59 |
|  | 10 | M | 57 |
|  | 11 | F | 70 |
|  | 12 | M | 85 |
| CM | 1 | M | 87 |
|  | 2 | F | 51 |
|  | 3 | F | 51 |
|  | 4 | M | 37 |
|  | 5 | M | 58 |
|  | 6 | F | 66 |
|  | 7 | F | 57 |
|  | 8 | M | 51 |
| CS | 1 | M | 80 |
|  | 2 | M | 84 |
|  | 3 | F | 46 |
|  | 4 | F | 56 |
|  | 5 | F | 42 |
|  | 6 | M | 50 |
|  | 7 | M | 43 |
|  | 8 | M | 59 |
|  | 9 | F | 52 |
|  | 10 | F | 65 |
