# Supplementary figures and images for: Effects of intestinal colonization by Clostridium difficile and Staphylococcus aureus on microbiota diversity in healthy individuals in China
Source: BMC Infect Dis. 2018 May 3;18:207. doi: 10.1186/s12879-018-3111-z (PMC5934869; doi:10.1186/s12879-018-3111-z)

## Slide 1
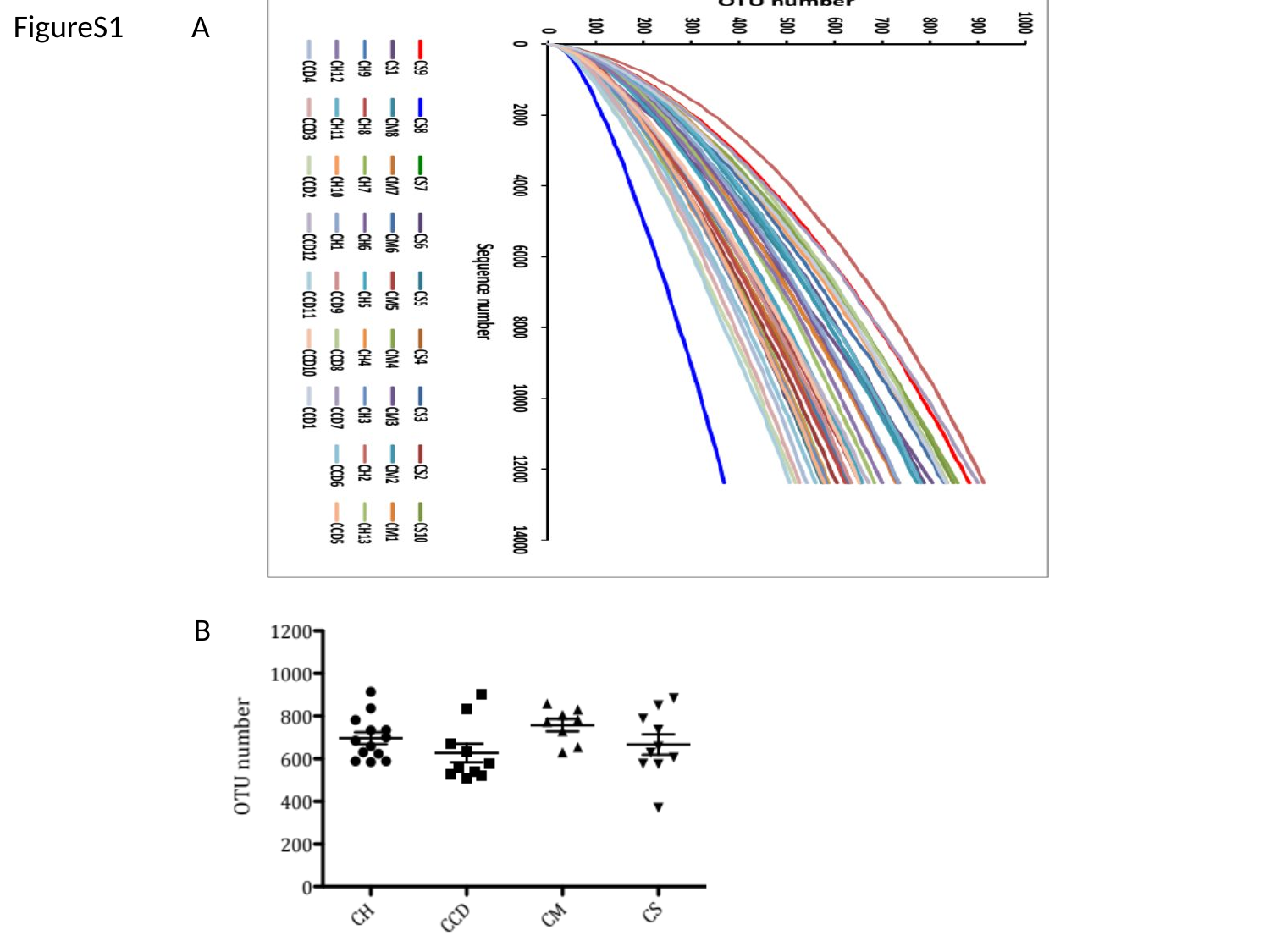

FigureS1
A
B

## Slide 2
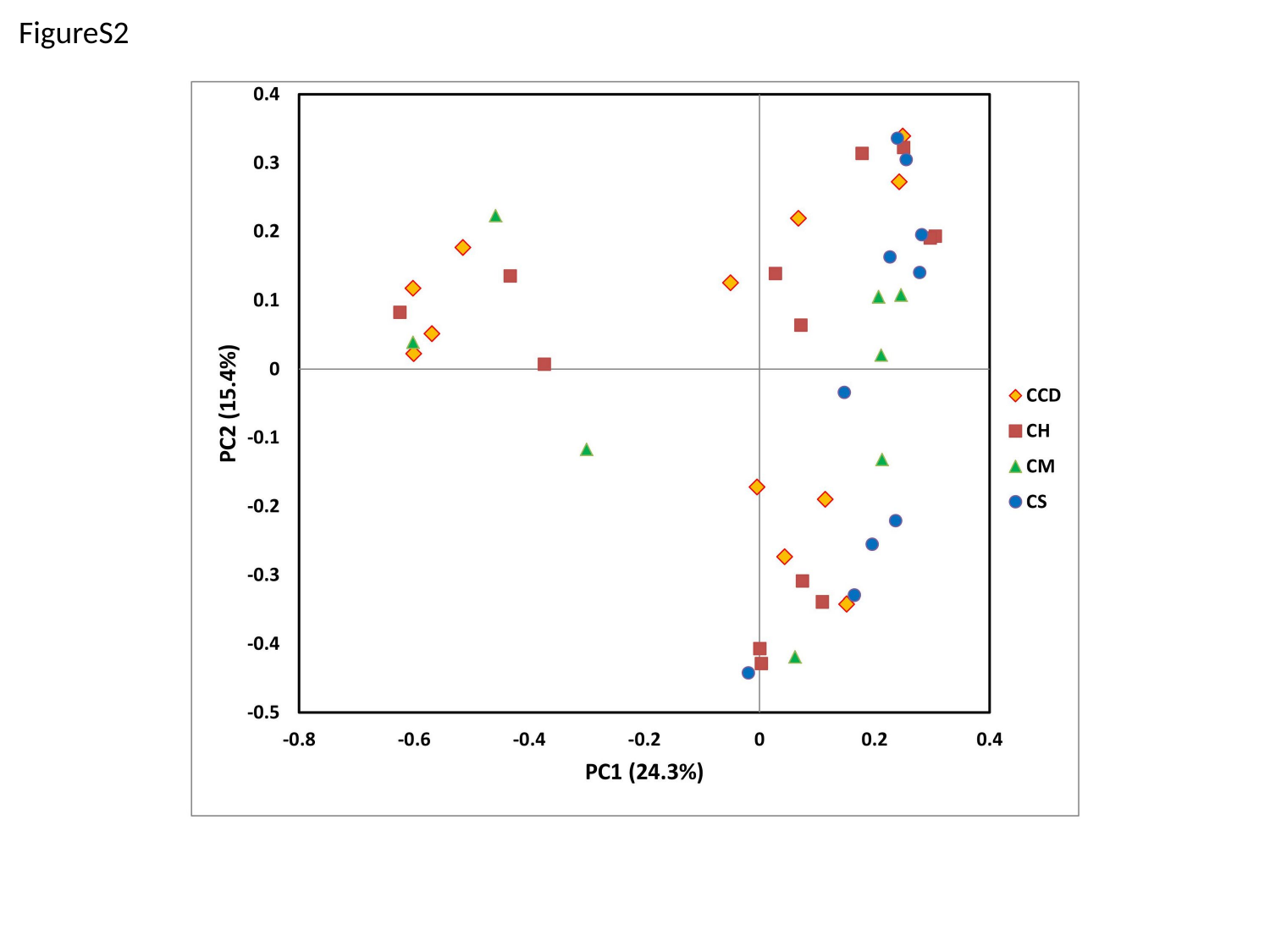

FigureS2

Supplement: Supplementary file 2 — Figure S1. Rarefaction curves for each sample and OTU numbers of each group. Figure S2. Principal coordinate analysis (PCoA) of bacterial communities using unweighted UniFrac distances of 16S rRNA gene sequences. (PPTX 521 kb) [file 12879_2018_3111_MOESM2_ESM.pptx]
